# Original Communications

**Published:** 1870-09

**Authors:** 


### *Original Communications.*

The completion of several valuable communications intended for the present No. has been prevented by the illness of the writers, of which we were not informed in season to obtain others. We have secured a corps of regular contributors that will usually keep our pages stocked with original matter.—All jokes, from whatever quarter, positively excluded. This out of deference to the American Mentor of medical journalism. However, that sufficient variety may be afforded to our readers, a reasonable latitude will be allowed correspondents. The use of calomel in *sedative* doses, bichromate of potash, and turpeth mineral in true croup, will not be considered infringing upon the rule against jokes.

